# Establishment of active chromatin structure at enhancer elements by mixed-lineage leukemia 1 to initiate estrogen-dependent gene expression

**DOI:** 10.1093/nar/gkt1236

**Published:** 2013-11-27

**Authors:** Kwang Won Jeong, Claudia Andreu-Vieyra, Jueng Soo You, Peter A. Jones, Michael R. Stallcup

**Affiliations:** ^1^Gachon Institute of Pharmaceutical Sciences, College of Pharmacy, Gachon University, Incheon 406-840, Republic of Korea, ^2^Departments of Urology and Biochemistry and Molecular Biology, USC Norris Comprehensive Cancer Center, Keck School of Medicine, University of Southern California, Los Angeles, CA 90089-9176, USA, ^3^Department of Biochemistry, School of Medicine, Konkuk University, Seoul 143-701, Republic of Korea and ^4^Department of Biochemistry and Molecular Biology, USC/Norris Comprehensive Cancer Center, Keck School of Medicine, University of Southern California, Los Angeles, CA 90089-9176, USA

## Abstract

A number of genome-wide analyses have revealed that estrogen receptor α binding to and regulation of its target genes correlate with binding of FOXA1, a pioneer factor, to nearby DNA sites in MCF-7 breast cancer cells. The enhancer element-specific histone H3K4me1/2 mark is enriched at the specific FOXA1/ERα recruitment sites in chromatin, but the mechanism by which these enhancer marks are established in chromatin before hormone treatment is unclear. Here, we show that mixed-lineage leukemia 1 (MLL1) protein is a key determinant that maintains permissive chromatin structure of the *TFF1* enhancer region. MLL1 occupies the *TFF1* enhancer region and methylates H3K4 before hormone stimulation. *In vitro*, MLL1 binds directly to the CpG-rich region of the *TFF1* enhancer, and its binding is dependent on hypomethylation of DNA. Furthermore, the depletion of MLL1 in MCF-7 cells results in a dramatic decrease of chromatin accessibility and recruitment of FOXA1 and ERα to the enhancer element. Our study defines the mechanism by which MLL1 nucleates histone H3K4 methylation marks in CpG-enriched regions to maintain permissive chromatin architecture and allow FOXA1 and estrogen receptor α binding to transcriptional regulatory sites in breast cancer cells.

## INTRODUCTION

Enhancers are distal *cis*-acting elements that regulate gene expression in a highly controlled manner. It is generally thought that the enhancer region is looped out or organized to permit enhancer–promoter interaction. This flexibility of enhancer element position relative to the transcription start site, however, impedes the comprehensive assignment of enhancer elements to the target genes they control. Recent genome-wide studies from independent laboratories have revealed several characteristic chromatin ‘signatures’ that define the enhancer elements in genes. For enhancer elements that are actively engaged or poised for activation, these signatures consist of high levels of p300 occupancy and H3K4me1 and a low level of H3K4me3 (1–4). A recent study of embryonic stem cells suggested three classes of enhancers: active (H3K4me1+, H3K27ac+), intermediate (H3K4me1+, H3K27−) and poised (H3K4me1+, H3K27me3+) (4,5). However, the mechanisms by which these histone marks are maintained and cooperate with other epigenetic factors, such as DNA methylation and pioneer factors, remain unclear.

Mixed lineage leukemia (MLL) proteins are SET1 family members that play critical roles in *HOX* gene activation (6–8). In mammals, six SET1 family members have been identified: SET1A, SET1B and four MLL family H3K4 methyltransferases, MLL1, MLL2, MLL3 and MLL4. Ash2, WDR5 and RBBP5 have been reported to form the core components of MLL1, MLL2, MLL3 and SET1A (7,9–11). Recent studies have also demonstrated that MLLs act as coactivators for nuclear receptor-mediated activation (12–15). Recently, we found that MLL1 plays a critical role in estrogen-dependent gene expression, possibly through the establishment of a histone methylation mark at the enhancer region of target genes (16), suggesting a potential role of MLL1 or other H3K4-specific histone methyltransferases in maintaining the local chromatin structure in the enhancer region.

The pioneer factor FOXA1 is a transcription factor that binds to thousands of sites across the human genome in breast and prostate cancer cells (17–19). FOXA1 is believed to scan chromatin for enhancers with forkhead motifs and can actively initiate chromatin decompaction (20–22). FOXA1 functions as a permissive or pioneer factor for estrogen receptor (ER) α-chromatin interactions and transcriptional activity in diverse target tissues (18). In particular, FOXA1 binds to genomic regions showing locally high levels of H3K4me1 and DNA hypomethylation, and its cell-type-specific recruitment to chromatin is inversely correlated with the differential DNA methylation levels of its corresponding binding sites (17,23). Selective DNA hypomethylation is also associated with the formation of DNase I hypersensitive chromatin regions (24–26) at potential glucocorticoid receptor (GR) binding sites, which are enriched in CpG dinucleotides compared with the surrounding genomic regions (27). Although these studies imply that DNA hypomethylation, H3K4me and FOXA1 binding are present in enhancer elements found within a pre-existing open chromatin structure, a comprehensive knowledge of the functional relationships among these enhancer marks is still lacking.

Considering that MLL1 binds to specific clusters of CpG residues within the *HOXA1* locus and regulates the expression of multiple transcripts (28), we hypothesized that MLL1 plays a role in maintaining the pre-existing open chromatin structure of ERα-target genes by establishing methylation marks. We determined that MLL1 binds directly to the CpG-rich region of the *TFF1* enhancer, and that its binding is dependent on DNA hypomethylation. Using formaldehyde-assisted isolation of regulatory elements (FAIRE)-qPCR to assess chromatin conformation and nucleosome occupancy methylome–sequencing (NOMe-seq) to monitor nucleosome positioning, we investigated how the depletion of MLL1 affects the establishment of open (active) chromatin and the binding of the pioneer factor, FOXA1. These findings provide the first evidence of the molecular mechanisms of MLL1, which nucleates histone H3K4 methylation marks at CpG islands to maintain chromatin accessibility for binding of ERα and its pioneer factor FOXA1 in breast cancer cells.

## MATERIALS AND METHODS

### Plasmids and cell culture

The pSG5–2 × FLAG expression vectors encoding the following proteins were constructed by inserting the appropriate cDNA coding region into EcoRI and XhoI sites: pSG5–2 × FLAG-MLL1(CX) (a.a. 1067–1432) and pSG5–2 × FLAG-MLL1 (CXPB) (a.a. 1067–1989). The pTriEX-His-MLL1(CX) expression vector encoding His-tagged MLL1 (a.a. 1067–1432) was constructed by inserting the appropriate cDNA coding region into NcoI and XhoI sites. Point mutations in MLL1 were introduced by QuikChange site-directed mutagenesis kit (Stratagene). pF-MLL1 was kindly provided by Jay Hess (University of Michigan). MCF-7, MDA-MB231 and 293T cells were maintained in Dulbecco’s modified Eagle’s medium supplemented with 10% fetal bovine serum.

### RNA interference and qRT-PCR

Small interfering RNA experiments were performed according to previously published methods (29). Transfection of the MCF-7 cells was performed with Oligofectamine (Invitrogen) according to the manufacturer’s protocol. The sequences for siRNAs are available in Supplementary data. Total RNA was isolated from MCF-7 cells with Trizol (Invitrogen) after hormone treatment as indicated and subjected to reverse transcription (RT) by iScript cDNA synthesis kit (Bio-Rad) in a total volume of 50 µl. In all, 2 µl of RT product was used for qPCR analysis with the primers shown in Supplementary data. Relative expression levels were normalized to GAPDH mRNA levels. Results shown are mean and range of variation of duplicate PCR reactions performed on the same cDNA sample; the results are from a single experiment that is representative of at least two independent experiments.

### Cell proliferation assay

MCF-7 cells were plated into six-well plates (2 × 10^5^ cells/well). The next day, the cells were transfected with siRNA and then cultured for 3 days in Dulbecco’s modified Eagle’s medium supplemented with 10% fetal bovine serum. Cells were trypsinized on indicated days, and total live cells were counted after Trypan blue staining.

### Chromatin immunoprecipitation (ChIP) assay

ChIP assays were performed according to previously described protocols (16,29). Briefly, MCF-7 cells were transfected with siRNA and then cultured for 3 days in phenol red-free Dulbecco’s modified Eagle’s medium supplemented with 5% charcoal-dextran-stripped fetal bovine serum. At ∼90% confluency, cells were treated with 100 nM estradiol (E2) or ethanol for the indicated time. After cross-linking with formaldehyde, the cell extracts were prepared from control and E2-treated MCF-7 cells. Immunoprecipitation of sonicated chromatin solutions was conducted by overnight incubation at 4°C with anti-ERα (Santa Cruz Biotechnology) anti-MLL1 (Bethyl Laboratories) or anti-FOXA1 (Abcam) antibody. Cross-linking was reversed by heating, and immunoprecipitated DNA was purified by phenol-chloroform extraction and ethanol precipitation. The purified DNA was dissolved in 100 µl of TE buffer [10 mM Tris–HCl (pH 8.0), 1 mM EDTA] and analyzed by quantitative PCR using Roche LightCycler 480 II system with SYBRGreen dye. For the Transfection-ChIP experiment, MCF-7 cells were transiently transfected with 2 × FLAG-tagged MLL1 for 3 days before E2 treatment. Immunoprecipitation was conducted with anti-FLAG antibody (Sigma Aldrich). Results shown are mean and range of variation of duplicate PCR reactions performed on the same DNA sample. Results were expressed as percentage of input chromatin (before immunoprecipitation) and were derived from a single experiment that is representative of at least two independent experiments.

### Bisulfite genomic sequencing

Genomic DNA was purified by phenol/chloroform extraction and ethanol precipitation. Bisulfite conversion was performed using the EZ DNA methylation kit (Zymo Research), and molecules were cloned using the Topo TA Kit (Invitrogen) according to the manufacturers’ instructions.

### DNA pull down assay and immunoblotting

FLAG-tagged MLL proteins were expressed in 293T cells, and total cell lysates were used for the binding assay. The 6 × His-tagged proteins were expressed in *Escherichia coli* strain BL21(DE3), purified by incubation with Ni-NTA agarose beads and eluted with phosphate-buffered saline (PBS) containing 250 mM imidazole. Point mutations in MLL1 were introduced by QuikChange site-directed mutagenesis kit (Stratagene). The CpG-rich enhancer (approximately −10 kb from TSS) or control region (approximately −5 kb from TSS) of the *TFF1* gene was amplified using 5′-biotinylated oligonucleotide primers. Biotinylated PCR products were immobilized on streptavidin-agarose beads and incubated with MLL1 proteins. Immunoblotting was performed as described previously (29), using the following antibodies: anti-His (Santa Cruz Biotechnology); anti-FLAG (Sigma Aldrich); anti-ERα, anti-tubulin (Santa Cruz Biotechnology); anti-MLL1 (Bethyl Laboratories); and anti-FOXA1 (Abcam).

### FAIRE-qPCR

FAIRE-qPCR was performed as previously described (30). Briefly, MCF-7 cells were transfected with siRNA and then cultured for 3 days in phenol red-free Dulbecco’s modified Eagle’s medium supplemented with 5% charcoal-dextran-stripped fetal bovine serum. At ∼90% confluency, the cells were treated with 100 nM E2 or ethanol for 60 min. After cross-linking with formaldehyde, cell extracts were prepared from control and E2-treated MCF-7 cells. Cross-linked chromatin was sonicated and open chromatin (FAIRE DNA) was purified from the aqueous phase by phenol-chloroform extraction and ethanol precipitation. For input control (Input DNA), cross-linking was reversed by heating before extraction. The purified DNA was dissolved in 100 µl of TE buffer and analyzed by the Roche LightCycler 480 II system with SYBRGreen dye. Results shown are mean and range of variation of duplicate PCR reactions performed on the same DNA sample. Results were expressed as percentage of input chromatin (Input DNA) and were derived from a single experiment that is representative of at least two independent experiments.

### Nucleosome occupancy methylome-sequencing (NOMe-seq)

NOMe-seq analysis of enhancers was performed as previously described (31), with minor modifications. Briefly, MCF-7 cells in 15-cm dishes were transfected with siRNA and then cultured for 3 days in phenol red-free Dulbecco’s modified Eagle’s medium supplemented with 5% charcoal-dextran-stripped fetal bovine serum. Chromatin was isolated from 200 000 cells and treated for 60 min with E2 or ethanol. Isolated chromatin was incubated for 7.5 min with 200 U of GpC-specific enzyme, M.CviPI (New England BioLabs), followed by another 7.5 min of incubation with 100 U of M.CviPI. Enhancer regulatory regions were PCR amplified from DNA and cloned using the TOPO TA cloning kit (Invitrogen). Colonies were screened for positive clones, and at least 15 positive clones per amplicon were sequenced per experiment. Accessible regions (green bubbles) and inaccessible regions (white bubbles, pink bar) were plotted as bubble charts. Statistical analyses (chi-square) of the ethanol and E2 treatments were performed for one GpC site every 100 bp. The percentage of inaccessible molecules was calculated for every GpC site in the amplicon [percentage of inaccessibility = (number of inaccessible molecules/total number of molecules) × 100], and the results were averaged over a distance of 100 bp and plotted.

## RESULTS

### MLL1 mediates ERα-dependent transcriptional activity

**Figure 1.**
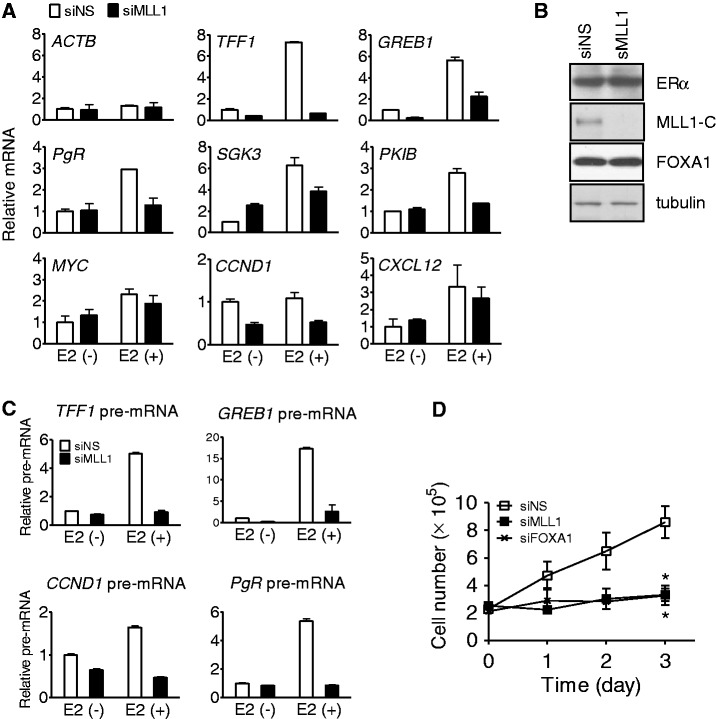
Requirement of MLL1 for the expression of endogenous ERα target genes and cell proliferation. (**A**) Effect of reduced MLL1 on the expression of estrogen-responsive genes. MCF-7 cells were transfected with siRNA against MLL1 (siMLL1) or non-specific siRNA (siNS), and treated with E2 (10 nM) for 16 h before harvest. Total RNA was analyzed by qRT-PCR. Levels of all mRNAs were normalized to that of *GAPDH* mRNA; β-actin mRNA served as a control that was unaffected by E2 or by siMLL1. (**B**) Depletion of MLL1 protein by siRNA transfection. MCF-7 cells were transfected with siMLL1 or siNS as in (A) and grown in hormone-free media for 72 h. Levels of ERα, tubulin and C-terminal cleavage of MLL1 (MLL1-C) were assessed by immunoblotting. (**C**) The pre-mRNA levels in the MCF-7 cells transfected with siNS or siMLL1 were measured by qRT-PCR using primers spanning the 3′ end of exon 1 and the 5′ end of intron 1 of each gene. Cells were treated for 8 h with 10 nM E2. Results shown are the mean and range of variation of duplicate PCR reactions performed on the same cDNA sample; the results are from a single experiment that is representative of at least two independent experiments. (**D**) Cell proliferation was performed in MCF-7 cells transfected with siNS or siMLL1 and then cultured for 3 days in normal media supplemented with 10% fetal bovine serum. Cells transfected with siFOXA1 were used as a positive control. The data are the mean of independent replicates ± S.D.

To understand the function of MLL1 in ERα-mediated transcription, we monitored the expression of several endogenous ERα target genes in MCF-7 breast cancer cells. Compared with cells transfected with non-specific siRNA (siNS), the estradiol (E2)-induced expression of *TFF1*, *GREB1*, *PgR*, *SGK3* and *PKIB* was compromised (Figure 1A) when MLL1 was depleted from MCF-7 cells by siRNA duplexes (Figure 1B and Supplementary Figure S1B). MLL1 cleavage by taspase generates N-terminal 320 kDa and C-terminal 180 kDa fragments, which form a stable complex that localizes to a subnuclear compartment (32). The levels of E2-induced *MYC* and *CXCL12* mRNAs were marginally reduced, and the basal expression of *CCND1* was affected by the depletion of MLL1, and thus MLL1 is critically required for E2-induced expression of most of the ERα target genes examined in this study.

We also measured the pre-mRNA levels of four ERα target genes, which is a more reliable reflection of the rapid changes in transcriptional rates, by using PCR primers spanning an exon/intron junction. E2 treatment increased the pre-mRNA levels of *TFF1*, *GREB1*, *CCND1* and *PgR* after 8 h, and most of this increase was eliminated by depletion of MLL1 (Figure 1C), indicating that MLL1 is critically involved in the transcriptional regulation of endogenous ERα target genes. Furthermore, silencing of endogenous MLL1 by siRNA in MCF-7 cells growing in normal media containing fetal bovine serum, which is equivalent to growth in E2, resulted in significant growth inhibition (*P* < 0.01) (Figure 1D), confirming that hormone-dependent expression of ERα-target genes and the growth of MCF-7 cells require MLL1. Similar results were obtained on the levels of mRNA (Supplementary Figure S1) and pre-mRNA (data not shown) of ERαtarget genes by using a second siRNA that targeted a different part of the MLL1 coding region.

### MLL1 is recruited to the hypomethylated CpG-rich enhancer region of the *TFF1* gene

**Figure 2.**
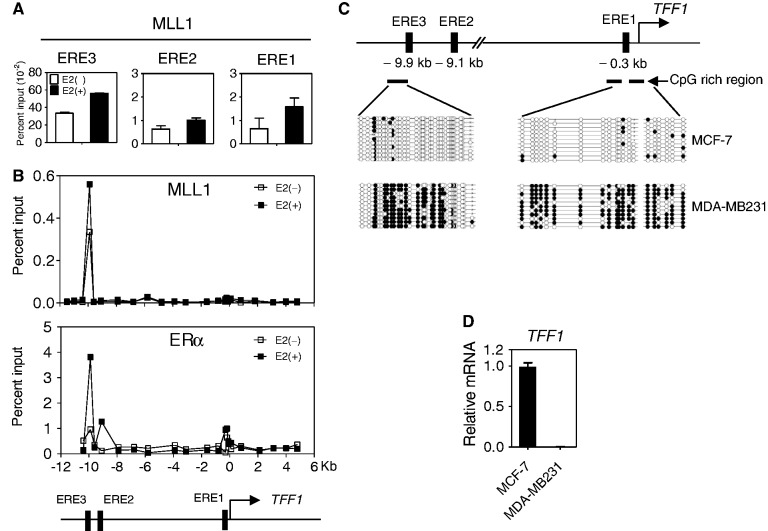
Recruitment of MLL1 to the hypomethylated CpG-rich enhancer region of the *TFF1* gene. (**A**) ChIP assays were performed with MCF-7 cells treated with E2 (100 nM) or ethanol for 1 h. The amount of the indicated regions surrounding the three *TFF1* ER binding sites precipitated by MLL1 antibody was determined by qPCR. Results shown are the mean and range of variation of duplicate PCR reactions performed on the same DNA sample. Results were expressed as percent of input chromatin (before immunoprecipitation) and were derived from a single experiment, which is representative of at least two independent experiments. (**B**) ChIP scanning of the *TFF1* locus. DNA precipitated with the indicated antibody was analyzed by qPCR with primer sets spaced at 1-kb intervals and spanning the region from −10 to +5 kb relative to the TSS (designated by a horizontal arrow in the diagram) of the *TFF1* gene. Primer sequences for ChIP scanning are available on request. (**C**) The *TFF1* gene displays cell type-specific methylation patterns. The DNA methylation of the *TFF1* promoter and enhancer regions in MCF-7 and MDA-MB231 cells was analyzed by bisulfite sequencing. Filled and open circles represent methylated and unmethylated CpG sites, respectively. Schematic diagrams of the *TFF1* promoter and enhancer regions are shown. CpG-rich regions were determined by CpG Island Searcher. (**D**) mRNA levels of *TFF1* was determined in MCF-7 and MDA-MB231 cells grown in normal media.

Although MLL1 is necessary for ERα-mediated transcription, the specific genomic regions occupied by MLL1 are not known. TFF1 has three EREs, and ERE3 at −9.9 kb upstream of transcriptional start site (TSS) is the major ER binding site and considered to be the major enhancer element. Our chromatin immunoprecipitation (ChIP) assay showed that the enhancer region of the *TFF1* gene (ERE3, −9.9 kb) was highly occupied by MLL1 and that the recruitment of MLL1 to this region was further enhanced after E2 treatment in MCF-7 cells (Figure 2A). We validated these findings by a ChIP scanning assay, which surveyed from −10 kb upstream through 5 kb downstream of the TSS of the *TFF1* gene. The distal enhancer region of *TFF1* was found to be highly occupied by MLL1. Importantly, the enhancer region of *TFF1* gene was occupied by MLL1 even before E2 treatment (Figure 2B).

Although MLL1 is capable of localizing to the enhancer region in the absence of E2 (Figure 2B), and has a CXXC motif that is known to bind to the unmethylated CpG region of the *HOXA9* gene (28,33), we investigated whether the enhancer region of *TFF1* contains hypomethylated CpG sites. The enhancer region to which MLL1 binding occurred has a high CpG content and is not methylated in MCF-7 cells that actively express the *TFF1* gene (Figure 2C and D). In contrast, the enhancer region of *TFF1* in MDA-MB231 cells, which are ERα-negative and do not express the *TFF1* gene, was highly methylated.

### MLL1 binds directly to the unmethylated CpG region of *TFF1* gene

**Figure 3.**
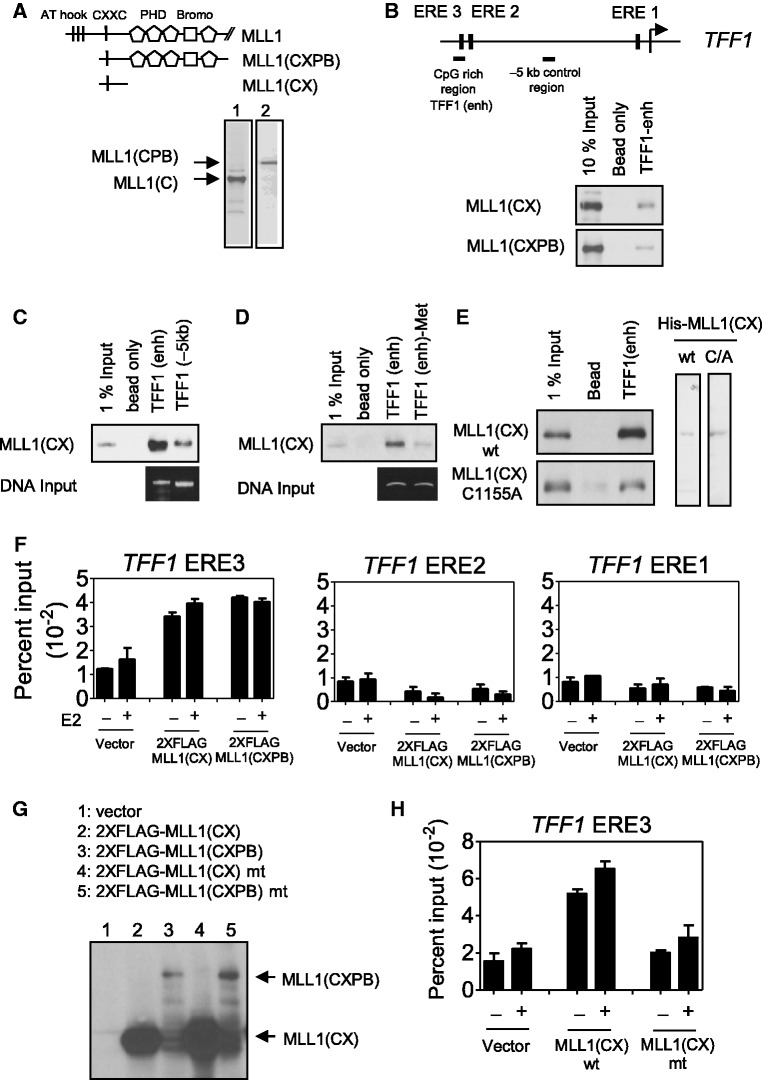
Direct interaction of MLL1 with the unmethylated CpG region of *TFF1* gene. (**A**) Expression of truncated MLL1 fragment containing CXXC motif only (MLL1-CX), or CXXC motif, three PHD domains and one Bromodomain (MLL1-CXPB) in 293T cells. (**B**) The DNA-pull down assay was performed using total cell lysate of 293T cells transfected with FLAG-tagged MLL1-CX or MLL1-CXPB and incubated with biotinylated genomic PCR product of the CpG-rich region of the *TFF1* enhancer region bound to streptavidin-Sepharose beads. Bound proteins were analyzed by immunoblotting with anti-FLAG antibody. (**C**) FLAG-tagged MLL1-CX was incubated with biotinylated genomic PCR product of the CpG-rich TFF1 enhancer region or the −5 kb control region bound to streptavidin-Sepharose beads. (**D**) The biotinylated CpG-rich enhancer region of *TFF1* gene was methylated *in vitro* using the CpG Methyltransferase (M.SssI), immobilized on streptavidin-agarose beads and then incubated with MLL1-CX proteins. (**E**) Recombinant wild-type (wt) MLL1-CX or MLL1-CX with mutation (Cys1155 to Ala) in CXXC motif were tested for binding to the biotinylated CpG-rich region of *TFF1* enhancer. (**F**) FLAG-tagged MLL1-CX or MLL1-CXPB was transiently expressed in the MCF-7 cells, which were then grown in hormone-free media for 48 h and then treated with 100 nM E2 for 1 h before performing the ChIP assay with anti-FLAG antibody. Precipitated DNA was analyzed by qPCR with primers representing the ER binding sites of the *TFF1* genes. (**G**) The expression of MLL1-CX or MLL1-CXPB in the MCF-7 cells was monitored by immunoblotting using antibodies against the FLAG epitope. (**H**) FLAG-tagged wild-type MLL1-CX or AXXC mutant was transiently expressed in the MCF-7 cells. After E2 treatment for 1 h, the ChIP assay was performed with anti-FLAG antibody. The precipitated DNA was analyzed by qPCR using primers representing the ERE3 site of the *TFF1* gene.

As the enhancer region of the *TFF1* gene occupied by MLL1 is hypomethylated, we also assessed the possible effects of the CXXC motif on MLL1 binding to the unmethylated *TFF1* enhancer region. We first identified which protein domain of MLL1 binds specifically to the unmethylated or methylated CpG region of the *TFF1* gene. The MLL1-CXPB fragment (amino acids 1067–1989; containing a CXXC motif, four PHD finger domains and a bromodomain) bound to the CpG-rich ERE3-region (Figure 3A and B). In addition, a 40-kDa N-terminal fragment of MLL1 that contained only the CXXC motif (MLL1-CX) was sufficient for binding to the enhancer. However, MLL1-CX bound much more weakly to the −5 kb control region where the CpG level was low (Figure 3C), suggesting that the interaction was CpG-specific. *In vitro* methylation of the CpG-rich region by the CpG-specific methyltransferase (M.SssI) inhibited most of the binding of MLL1-CX (Figure 3D). Mutation of the CXXC motif of MLL1-CX to AXXC (Cys-1155 changed to Ala) eliminated most of the binding to the enhancer of the *TFF1* gene (Figure 3E), indicating that the CXXC motif is involved in the interaction between MLL1 and the unmethylated *TFF1* enhancer. When FLAG-tagged MLL1-CX or MLL1-CXPB was overexpressed in MCF-7 cells by transient transfection, quantitative ChIP assays performed with anti-FLAG antibody demonstrated the occupancy by both MLL1-CX and MLL1-CXPB on ERE3 (but not ERE2 or ERE1) of the *TFF1* gene, indicating that the 40 kDa MLL1-CX fragment is sufficient for recruitment to the enhancer region *in vivo* (Figure 3F) with the same genomic binding specificity as wild-type MLL1 (Figure 2B). Although both MLL1-CX and MLL1-CXPB showed similar occupancy to ERE3 (Figure 3F), the expression level of MLL1-CX was higher than that of MLL1-CXPB (Figure 3G), suggesting that MLL1-CXPB molecules occupy ERE3 more efficiently than MLL1-CX molecules, probably due to extra DNA interaction or structural stabilization through other domains. Furthermore, when FLAG-tagged wild-type or mutant MLL1-CX were overexpressed at similar levels in MCF-7 cells, only wild-type MLL1-CX, but not mutant MLL1-CX, was associated with the CpG-rich region of the *TFF1* enhancer (Figure 3H). Thus, these results indicate that the interaction of CXXC motif with the unmethylated CpG-rich enhancer region of the *TFF1* gene is required for MLL1 occupancy.

### MLL1 is required for binding of FOXA1 and ERα to the *TFF1* enhancer

**Figure 4.**
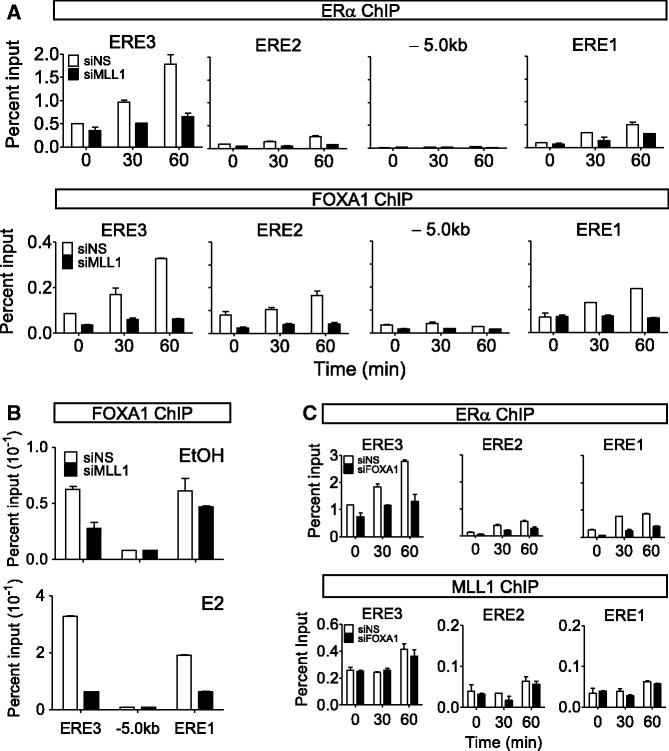
Effect of MLL1 depletion on the recruitment of ERαand FOXA1 to *TFF1* EREs. (**A-C**) ChIP assays were performed as detailed in Figure 2 after transfection with siNS, siMLL1 or siFOXA1. The MCF-7 cells were treated with E2 (100 nM) or ethanol for the indicated times (A, C) or 60 min (B). The amount of the indicated region of the *TFF1* gene precipitated by ERα, MLL1 or FOXA1 antibodies was determined by qPCR.

In MCF-7 cells, H3K4me1 and me2 are detected at estrogen responsive enhancers before E2 stimulation and ERα binding, reminiscent of FOXA1 recruitment (17). We have also shown that MLL1 occupies ERE3 of *TFF1* before estrogen treatment (Figure 2A) and is responsible for the histone H3K4 methylation marks at the promoter and enhancer regions of the *TFF1* gene before E2 treatment (16). We hypothesized that MLL1 may positively regulate the binding of FOXA1 by establishing the H3K4me signature at the enhancer region of *TFF1*. To investigate whether MLL1 is required for ERα and FOXA1 binding, we assessed their occupancies at EREs in MLL1-depleted MCF-7 cells. Depletion of MLL1 caused dramatic decreases in the occupancy of ERα and FOXA1 at ERE3 (Figure 4A), indicating that both ERα and FOXA1 recruitment are dependent on MLL1. Similar results were obtained by using a second siRNA that targeted a different part of the MLL1 coding region (Supplementary Figure S2).

In the absence of E2, FOXA1 occupancy at ERE3 was 7.8-fold higher than that in the −5 kb control region, and increased 5.2-fold after estrogen treatment in MCF-7 cells. Surprisingly, specific silencing of MLL1 resulted not only in a 2-fold decrease of the pre-existing FOXA1 occupancy (Figure 4B, upper panel) but also in a dramatic inhibition of E2-induced FOXA1 recruitment (Figure 4B, lower panel), confirming the absolute requirement of MLL1 for the binding of FOXA1 to ERE3 of the *TFF1* gene. We validated these findings in independent experiments (Supplementary Figure S3). In contrast, depletion of FOXA1 by specific siRNA did not affect MLL1 occupancy of *TFF1* ERE3 (Figure 4C lower panel and Supplementary Figure S4B), even though E2-induced ERα binding to ERE3 was almost eliminated (Figure 4C, upper panel and Supplementary Figure S4A). These data support the notion that MLL1 is the key molecule that regulates chromatin occupancy by FOXA1 both before and after E2 stimulation, and also regulates the subsequent E2-induced ERα recruitment to the *TFF1* enhancer.

### Role of MLL1 in chromatin accessibility

**Figure 5.**
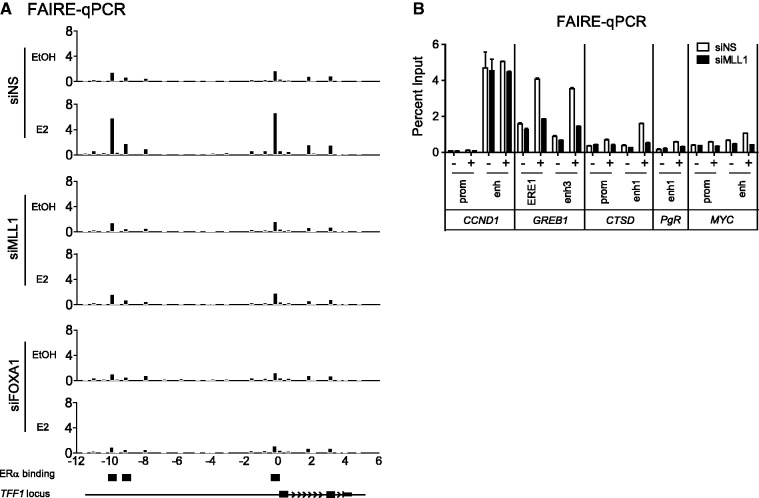
MLL1 is required for open chromatin structure. (**A**) Chromatin accessibility at the *TFF1* locus was assessed by FAIRE-qPCR analysis using chromatin samples prepared from the MCF-7 cells transfected with siNS, siMLL1 or siFOXA1. Data are normalized against non–cross-linked genomic DNA for each primer pair. (**B**) The FAIRE-qPCR analysis was performed at the promoter and enhancer regions of *CCND1*, *GREB1*, *CTSD*, *PgR* and *MYC* in MLL1-depleted or control MCF-7 cells treated with E2 or ethanol for 60 min.

Given that the enhancer region of the *TFF1* gene requires MLL1 for the binding of FOXA1 and ERα and for transcriptional activity (Figures 1 and 4), we investigated whether MLL1 influences the chromatin accessibility of the enhancer region of the *TFF1* gene. We performed formaldehyde-assisted isolation of regulatory elements (FAIRE) coupled with qPCR. Hormone-deprived MCF-7 cells were transfected with siNS or siMLL1 and treated with ethanol or E2 for 1 h. siNS-transfected and ethanol-treated MCF-7 cells showed weak FAIRE signals at the proximal and distal ERα binding sites (Figure 5A). We observed a several-fold increase in the FAIRE signal after estrogen treatment at the ERα binding sites (ERE1 and 3). Surprisingly, specific silencing of MLL1 by siRNA dramatically decreased the E2-induced FAIRE signal of the enhancer and promoter regions of the *TFF1* gene that were observed in siNS-transfected cells (Figure 5A), suggesting reduced chromatin accessibility to EREs caused by MLL1 depletion. The silencing of FOXA1 also reduced the estrogen-induced FAIRE signal. Similarly, depletion of MLL1 altered the chromatin accessibility of the promoter and enhancer regions of other ERα-mediated genes (*GREB1, CTSD, PgR* and *MYC*) (Figure 5B). In addition, ERα loading onto the enhancer region of these genes appears to be correlated with MLL1 occupancy. For example, ERα binding to *TFF1, GREB1, CTSD* and *MYC* is dependent on MLL1 (Supplementary Figure S5); in contrast, ERα binding to the *c11orf49* gene that is not occupied by MLL1 was not affected by MLL1 depletion. These data suggest that MLL1 is required to maintain the optimal chromatin structure at the enhancers of many estrogen target genes.

### Enhancer chromatin structure is maintained by MLL1

**Figure 6.**
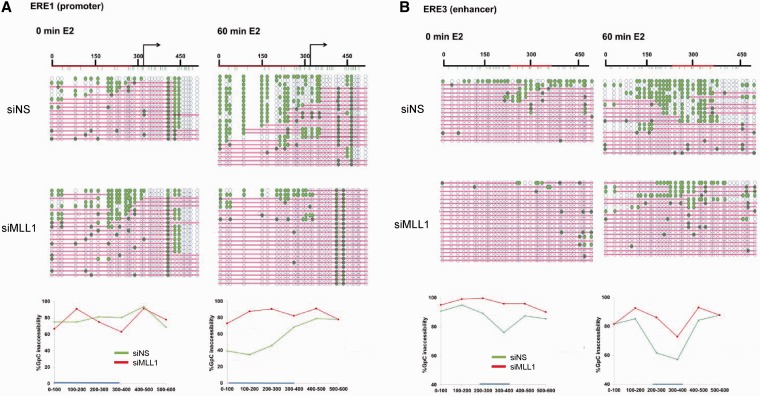
Nucleosome occupancy of the *TFF1* enhancer is regulated by MLL1. (**A** and **B**) Nucleosome occupancy at the *TFF1* promoter and enhancer was analyzed by NOMe-seq in siNS transfected or MLL1-depleted MCF-7 cells. Blue circles represent GpC sites of the DNA (unfilled blue circles represent GpC sites that are inaccessible to GpC methyltransferase; teal-filled circles represent cytosines accessible to GpC methyltransferase). Pink bars represent regions of inaccessibility large enough to accommodate a nucleosome (∼150 bp).

Although MLL1 is localized to the *TFF1* enhancer even before hormone stimulation, we were unable to observe any significant changes in the FAIRE signal by MLL1 silencing in ethanol-treated cells. A possible explanation for this may be the limited resolution level of the assay. To further understand the local nucleosome dynamics, we performed NOMe-seq, a method that measures the methylation of GpC in isolated chromatin by M.CviPI DNA methyltransferase, which allows high-resolution determination of nucleosome occupancy (Figure 6), as this enzyme only methylates DNA that is unoccupied by nucleosomes and other proteins. Although the region upstream of the TSS of the *TFF1* promoter is largely occupied by nucleosome (indicated by pink bars showing regions inaccessible to the GpC methyltransferase), it is somewhat accessible to enzyme (teal circles indicate methylation by the M.CviPI enzyme), indicating the presence of a minimal nucleosome-depleted region (NDR) in some of the cells that may allow the binding of basal transcription factors (Figure 6A). After 1 h of estrogen treatment, the *TFF1* promoter region is open more broadly and is open in a majority of the cells, generating a large NDR at the regulatory site of the ERα target gene. Similarly, a significant increase in accessibility was observed at the enhancer region after 60 min of E2-treatment (Figure 6B), suggesting that the increase of the NDR at both the promoter and enhancer regions is important for gene expression. However, in MLL1-depleted cells, the majority of the promoter region remained closed even after estrogen stimulation, and the estrogen-induced increase of the NDR at the enhancer region was remarkably reduced (Figure 6B), confirming that MLL1 appears to be important for hormone-induced nucleosome repositioning and chromatin accessibility. Importantly, NOMe-seq data in estrogen-starved MCF-7 cells revealed a substantial decrease in chromatin openness at the enhancer (ERE3) region in MLL1-depleted cells compared with control (siNS) cells (Figure 6B), indicating a role for MLL1 in facilitating active chromatin architecture prior to E2 treatment. Together, these data indicate that MLL1 plays a critical role in maintaining nucleosome positioning and chromatin configurations, which allow for establishment of active chromatin states required for gene activation.

## DISCUSSION

### Site-specific binding of MLL1 to *TFF1* enhancer

Activation or enhancement of transcription in response to a signal apparently involves scores or perhaps even hundreds of coregulator proteins that are recruited by the DNA-bound transcriptional activator protein and subsequently modulate local chromatin conformation and assembly and/or activation of a transcription complex involving RNA polymerase II. Most recently, the Encyclopedia of DNA Elements (ENCODE) project has systematically mapped regions of transcription, chromatin structure and histone modification, providing new insights into the mechanisms of gene regulation (http://encodeproject.org). One of the most highly studied gene systems is the *TFF1* gene and its response to estradiol; the timing of specific occupancy by many coregulators and histone modifications has been carefully documented (34,35). However, the mechanisms by which the highly choreographed sequence of events is coordinated are unknown. Here, we defined the role of MLL1, an H3K4-specific histone methyltransferase, in maintaining the local chromatin structure of an enhancer that is required for transcription initiation. We demonstrated how CpG hypomethylation, histone H3K4 methylation, FOXA1 binding and ERα recruitment are coordinated.

The CXXC motif is found in proteins that recognize the cytosine methylation status in genomic regions containing CpG dinucleotides; examples are DNMT1, methyl-CpG-binding protein (MBD), and human CpG-binding protein (hCGBP) (36–40). In MLL1, the CXXC motif binds to the unmethylated promoter of the *HOX* gene and regulates its expression (28,33,41). We have shown that the CXXC motif in MLL1 can also bind directly to the hypomethylated enhancer region of the *TFF1* gene (Figure 3). Introducing a mutation into the CXXC motif revealed that the DNA binding function of the CXXC motif is indispensable for MLL1 recruitment to the *TFF1* gene (Figure 3E and H). Previously, multiple MLL1 domains were shown to be necessary for the recruitment of MLL1 to the *HOXA9* gene *in vivo* (6). A fragment containing the CXXC region alone or PHD fingers 1–4 alone failed to associate with *HOXA9* in *Mll*^−^^/^^−^ mouse embryonic fibroblast cells. In contrast, we observed that the CXXC domain alone (MLL1-CX) was sufficient to bind to the CpG region of the *TFF1* gene *in vitro* and *in vivo*, although the inclusion of PHD fingers and bromodomain further increased MLL1 binding efficiency (Figure 3F and G). The binding of MLL1 to the enhancer appears to be guided by hypomethylated CpG. MLL1-CX does not bind efficiently to the −5 kb control region in which CpG content is low (Figure 3D), and *in vitro* methylation of the CpG-rich *TFF1* enhancer fragment by the CpG Methyltransferase (M.SssI) reduced the binding of MLL1-CX (Figure 3D). Thus, the interaction of the CXXC motif in MLL1 with the unmethylated CpG-rich enhancer region of the *TFF1* gene is required for occupancy by MLL1 (Figure 3); and the MLL1 occupancy of the ER binding sites associated with many ER target genes that require MLL1 for E2-induced expression (Figure 2 and Supplementary Figure S5) suggests that MLL1 occupancy is required for the transcriptional activity of MLL1 in ERα-mediated gene expression (Figure 1).

### Requirement of MLL1 for ERα and FOXA1 binding

Previous studies have shown that FOXA1 is colocalized to the majority of ERα binding sites in MCF-7 cells (17,19). Moreover, introduction of FOXA1 into U2OS cells that stably express ERα alters the ERα binding pattern (18), suggesting that one of the primary roles of FOXA1 is to aid ERα binding to chromatin (42). In addition, significant enrichment of histone H3K4me1/2 marks has been observed at the FOXA1 recruitment site (17). Differential binding of FOXA1 to chromatin is dependent on the distribution of H3K4 dimethylation, although how H3K4me1/2 guides FOXA1 recruitment remains uncertain (17). Recently, it was also reported that FOXA1 binds to genomic regions showing local DNA hypomethylation, and histone H3K4 methylation at the enhancers may stabilize FOXA1 binding during P19 cell neural differentiation (23,43). Importantly, during cellular differentiation, activation of FOXA1-dependent enhancers is accompanied by a decrease in DNA methylation (23), suggesting a strong correlation between DNA hypomethylation and FOXA1 binding.

In this study, we showed that both FOXA1 and ERα binding required MLL1, which preferentially occupies the CpG-rich region of the *TFF1* enhancer region (Figure 4). Knockdown of MLL1 resulted in dramatic decreases in ERα and FOXA1 occupancy at ERE3 (Figure 4A). Given that MLL1 occupies the enhancer regions of *TFF1* and other E2-induced genes before estrogen treatment (Figure 2A and Supplementary Figure S5) and establishes the histone H3K4 methylation marks (16), our results indicate that MLL1 is a key molecule that regulates chromatin occupancy by FOXA1 by maintaining the histone methylation mark in the absence of E2. Thus, MLL1 facilitates binding of ERα after E2 stimulation by maintaining permissive chromatin architecture that allows FOXA1 occupancy.

Interestingly, FOXA1 occupancy at the enhancer of *TFF1* was increased by ∼5.2-fold after E2 stimulation. The most likely explanation is that chromatin remodeling triggered by the SWI/SNF or other chromatin remodeling complexes facilitated the further occupancy of FOXA1 on its binding site. Similarly, the increase of FOXA1 occupancy by E2 has been observed at several ERα binding sites (ERBS-1, ERBS-3, ERBS-5) in the *RET* gene locus (44). Another possible explanation for this observation may be that FOXA1 became more accessible to the ChIP antibody after E2 stimulation through the formation of relaxed open chromatin structure caused by SWI/SNF.

### Role of MLL1 in the chromatin structure of *TFF1* enhancer

Hormone-deprived MCF-7 cells showed weak FAIRE signals at proximal and distal ERα binding sites (Figure 5A), but were somewhat accessible to the M.CviPI enzyme, suggesting a minimal NDR that may allow for the binding of basal transcription factors. The FAIRE signal increased significantly after estrogen treatment at ERα binding sites (ERE1–3). Thus, estrogen binding sites (ERE1–3) seem to be partially opened in the absence of E2 (Figures 5A and 6). Surprisingly, specific silencing of MLL1 by siRNA dramatically inhibited the E2-induced increases in the FAIRE-signal and M.CviPI DNA methyltransferase accessibility of the enhancer and promoter regions of the *TFF1* gene compared with siNS-transfected cells (Figures 5A and 6).

The correlation between the presence of a hypomethylated CpG region and chromatin hypersensitivity has been previously reported (45–48). Most recently, it was proposed that DNA methylation status may also play a role in the formation of open chromatin regions and in the maintenance of enhancer elements (27,49,50). Cells create and maintain a pre-programmed accessible chromatin architecture at active and potentially active transcriptional regulatory sites that includes specific histone modifications and, at many sites, is also characterized by enriched-CpG hypomethylation. However, the mechanism by which the transcriptionally permissive chromatin architecture (including H3K4 methylation) at DNA hypomethylation sites is maintained remains unclear. We demonstrate here that both the estrogen-induced increase of NDR extent and frequency at enhancer regions and the basal chromatin accessibility were remarkably reduced by MLL1 depletion. These results suggest that MLL1 plays a critical role in maintaining nucleosome positioning as well as epigenetic marks and possibly other aspects of chromatin configurations before and after hormone stimulation, thus facilitating the establishment of active chromatin states by H3K4 methylation in MCF-7 breast cancer cells. It is not clear to us at this time how the H3K4 methylation by MLL1 coordinates FOXA1 binding to *TFF1* enhancer, but much work remains to be done to understand the relationships among epigenetic marks, chromatin architecture, nucleosome positioning and occupancy by transcription factors at enhancer elements. However, the observation that the depletion of MLL1 reduced pre-existing FOXA1 occupancy serves as an intriguing finding for further investigation.

## SUPPLEMENTARY DATA

Supplementary Data are available at NAR Online.

## FUNDING

National Institutes of Health (NIH) [DK043093 to M.R.S., R37CA 082422 to P.A.J.]; National Cancer Institute provided the Cancer Center Support Grant [P30CA014089 to the University of Southern California]. Funding for open access charge: NIH [DK043093].

*Conflict of interest statement*. None declared.

## Supplementary Material

Supplementary Data
