# Supplementary material for: Establishment of active chromatin structure at enhancer elements by mixed-lineage leukemia 1 to initiate estrogen-dependent gene expression
Source: Nucleic Acids Res. 2013 Nov 27;42(4):2245–56. doi: 10.1093/nar/gkt1236 (PMC3936730; doi:10.1093/nar/gkt1236)
Supplement: Supplementary Data [file supp_42_4_2245__index.html]

Establishment of active chromatin structure at enhancer elements by mixed-lineage leukemia 1 to initiate estrogen-dependent gene expression — Establishment of active chromatin structure at enhancer elements by mixed-lineage leukemia 1 to initiate estrogen-dependent gene expression — Supplementary Data 

# Establishment of active chromatin structure at enhancer elements by mixed-lineage leukemia 1 to initiate estrogen-dependent gene expression

## Supplementary Data

files

**Files in this Data Supplement:**

- Supplementary Data - pdf file
