# Supplementary material for: Establishment of active chromatin structure at enhancer elements by mixed-lineage leukemia 1 to initiate estrogen-dependent gene expression
Source: Nucleic Acids Res. 2013 Nov 27;42(4):2245–56. doi: 10.1093/nar/gkt1236 (PMC3936730; doi:10.1093/nar/gkt1236)
Supplement: Supplementary Data [file supp_gkt1236_nar-02667-x-2013-File008.pdf]

Establishment of active chromatin structure at enhancer elements by mixed-lineage leukemia 1 to initiate estrogen-dependent gene expression

Kwang Won Jeong[2], Claudia Andreu-Vieyra[3], Jueng Soo You[4], Peter A. Jones[3], Michael R. Stallcup[1]

[1]Department of Biochemistry and Molecular Biology, USC/Norris Comprehensive Cancer Center, Keck School of Medicine, University of Southern California, Los Angeles, CA 90089-9176.
[2]Gachon Institute of Pharmaceutical Sciences, College of Pharmacy, Gachon University, Incheon 406-840, Republic of Korea
[3]Departments of Urology and Biochemistry and Molecular Biology, USC Norris Comprehensive Cancer Center, Keck School of Medicine, University of Southern California, Los Angeles, CA 90089-9176.
[4]Department of Biochemistry, School of Medicine, Konkuk University, Seoul, 143-701, Republic of Korea

Supplementary data

Inventory of Supplementary data

1. Supplementary Fig. 1, Requirement of MLL1 for the expression of endogenous ERα target genes.
2. Supplementary Fig. 2, Effect of MLL1 depletion on the recruitment of ERα and FOXA1 to TFF1 EREs
3. Supplementary Fig. 3, Effect of MLL1 depletion on the recruitment of FOXA1 in the absence of E2
4. Supplementary Fig. 4, Effect of FOXA1 depletion on the recruitment of ERα or MLL1.
5. Supplementary Fig. 5, ERα binding to *TFF1*, *GREB1*, *CTSD*, and *MYC* is dependent on MLL1.

Fig. S1

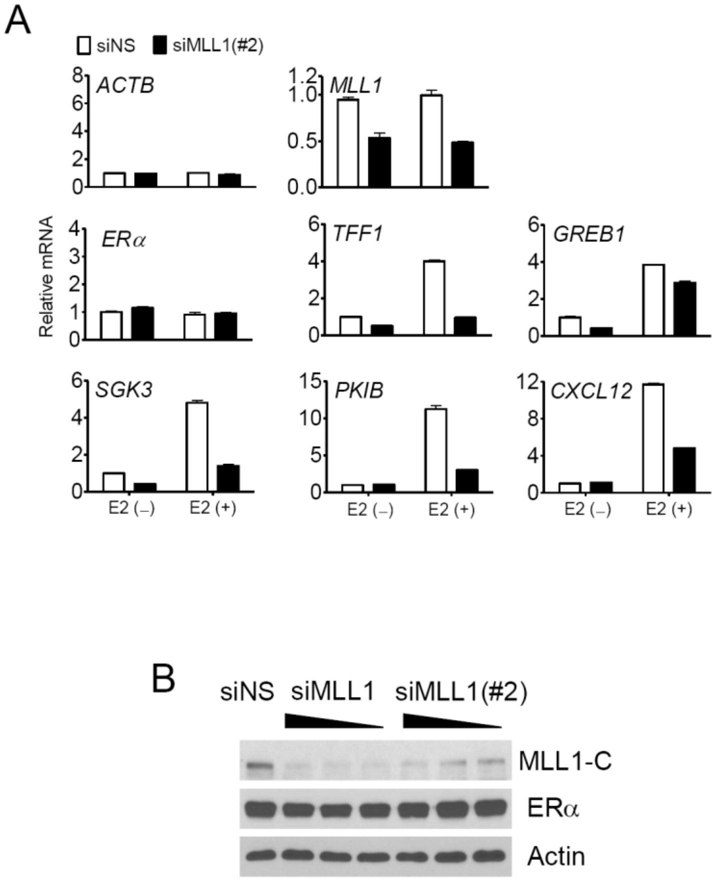

Supplementary Figure 1. Requirement of MLL1 for the expression of endogenous ERα target genes. (A) Effect of reduced MLL1 on the expression of estrogen-responsive genes. MCF-7 cells were transfected with siMLL1(#2) (which has a different target sequence from siMLL1) or non-specific siRNA (siNS), and treated with E2 (10 nM) for 16 hours before harvest. Total RNA was analyzed by qRT-PCR. Levels of all mRNAs were normalized to that of GAPDH mRNA. (B) Depletion of MLL1 protein by siMLL1 or siMLL1(#2) transfection. MCF-7 cells were transfected with siMLL1, siMLL1(#2) or siNS as in (A) and grown in hormone free-media for 72 h. Levels of ERα, β-actin and C-terminal cleavage product of MLL1 (MLL1-C) were assessed by immunoblotting.

Fig. S2

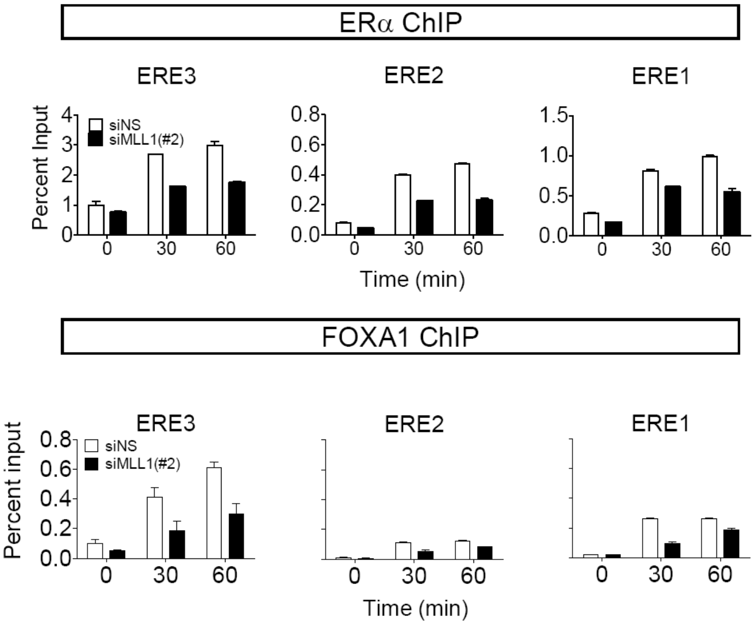

Supplementary Figure 2. Effect of MLL1 depletion on the recruitment of ERα and FOXA1 to *TFF1* EREs. ChIP assays were performed as detailed in Figure 2 after transfection with siNS or siMLL1(#2). The MCF-7 cells were treated with E2 (100 nM) or ethanol for 60 min. The amount of the indicated region of the *TFF1* gene precipitated by ERα or FOXA1 antibodies was determined by qPCR.

Fig. S3

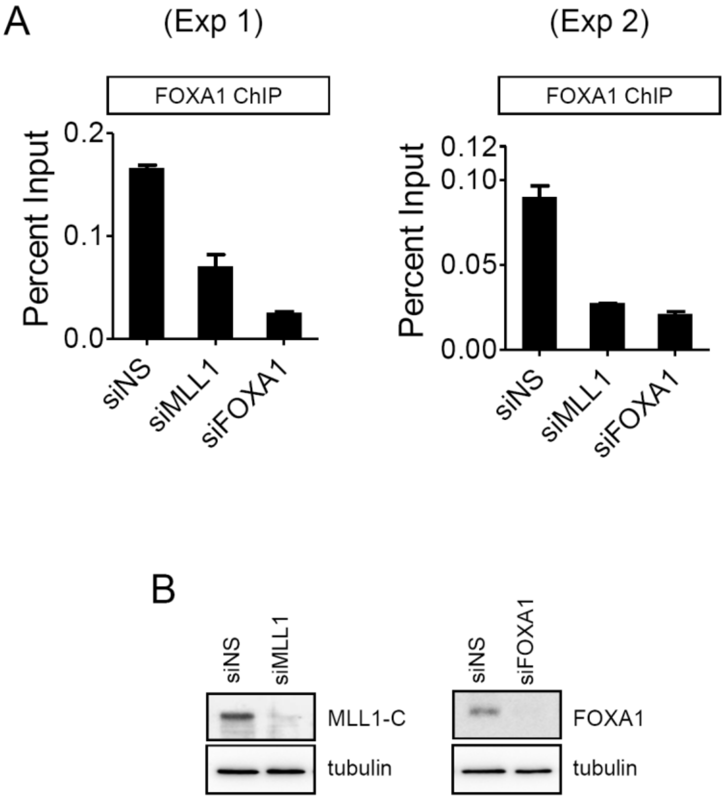

Supplementary Figure 3. Effect of MLL1 depletion on the recruitment of FOXA1 in the absence of E2. *(A)* ChIP assays were performed as detailed in Figure 2 after transfection with siNS , siMLL1 or siFOXA1. The MCF-7 cells were treated with ethanol for 60 min. The amount of the ERE3 region of the TFF1 gene precipitated by FOXA1 antibodies was determined by qPCR. (B) Depletion of MLL1 or FOXA1 protein by siMLL1 or siFOXA1 transfection. MCF-7 cells were transfected with siNS, siMLL1 or siFOXA1 as in (A) and grown in hormone free-media for 72 h. Levels of tubulin, FOXA1, and C-terminal cleavage of MLL1 (MLL1-C) were assessed by immunoblotting.

Fig. S4

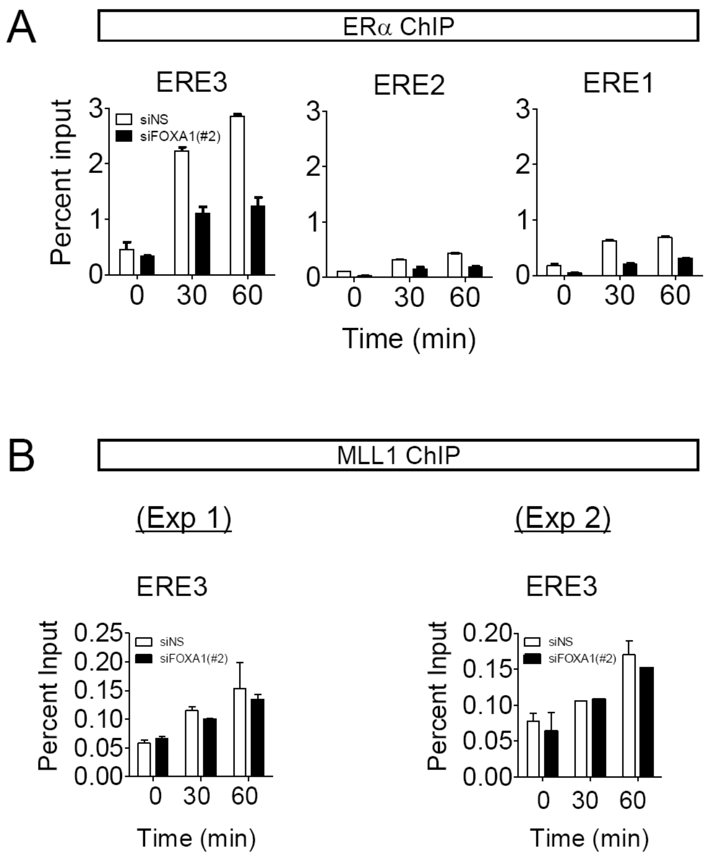

Supplementary Figure 4. Effect of FOXA1 depletion on the recruitment of ERα or MLL1. *(A-B)* ChIP assays were performed as detailed in Figure 2 after transfection with siNS or siFOXA1(#2). The MCF-7 cells were treated with E2 (100 nM) for the indicated time periods. The amount of the indicated region of the *TFF1* gene precipitated by ERα or MLL1 antibodies was determined by qPCR. *(B)* Data shown from two independent experiments

Fig. S5

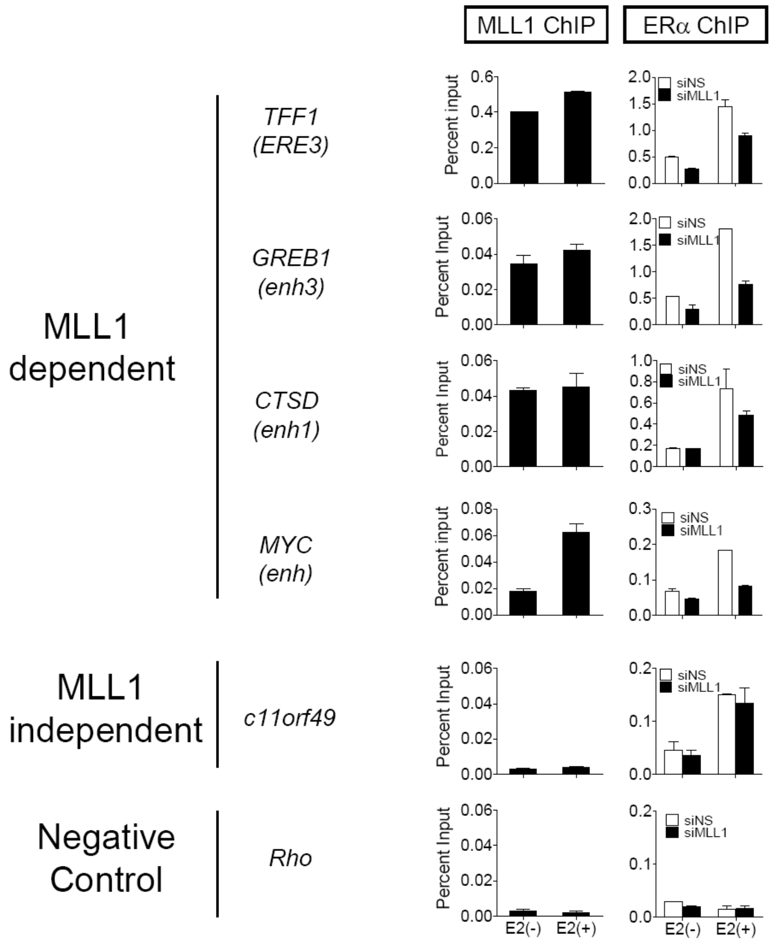

Supplementary Figure 5. ERα binding to *TFF1*, *GREB1*, *CTSD*, and *MYC* is dependent on MLL1. ChIP assays were performed as detailed in Figure 2 after transfection with siNS or siMLL1. The MCF-7 cells were treated with E2 (100 nM) or ethanol for 60 min. The amount of the indicated region of the *TFF1*, *GREB1*, *CTSD*, *MYC*, *c110rf49* and *Rho* gene precipitated by ERα or MLL1 antibodies was determined by qPCR.

Primer sequences for ChIP experiment

TFF1 (ERE1)
5'-GGCAGGCTCTGTTTGCTTAAAGAGCG-3' (forward)
5'-GGCCATCTCTCACTATGAATCACTTCTGC-3' (reverse)

TFF1 (ERE2)
5'-CCTCCCCAGCTCACGTTGT-3' (forward)
5'-GGGTTGCATTTAAGGGACCTT-3' (reverse)

TFF1 (ERE3)
5'-GTCGTTGCCAGCGTTTCC-3' (forward)
5'-CTTCTCCACGCCCTGTAAATTT-3' (reverse)

TFF1 (-4.8 kb)
5'-CGGTGGCCAGAGAGCATAA-3' (forward)
5'-CCCTTCGGGTGCCACTCAA-3' (reverse)

Primer sequences for qRT-PCR

ACTB
5'-ACCCCATCGAGCACGGCATCG-3' (forward)
5'-GTCACCGGAGTCCATCACGATG-3' (reverse)

TFF1
5'-GAACAAGGTGATCTGCG-3' (forward)
5'-TGGTATTAGGATAGAAGCACCA-3' (reverse)

GREB1
5'-CAAAGAATAACCTGTTGGCCCTGC-3' (forward)
5'-GACATGCCTGCGCTCTCATACTTA-3' (reverse)

PgR
5'-GTGCCTATCCTGCCTCTCAATC-3' (forward)
5'-CCCGCCGTCGTAACTTTCG-3' (reverse)

SGK3
5'-TGAGGCCAGGAGTGAGTCTT-3' (forward)
5'-TATCATCTGGTCCAGCAACA-3' (reverse)

PKIB
5'-CCAATTTTGCATCTTCAGCA-3' (forward)
5'-GGCTTTTCCAATTGGTCTTG-3' (reverse)

MYC

5’-CTCTCAACGACAGCAGCTCG-3’ (forward)
5’-CAACATCGATTTCTTCCTCATCTTC-3’ (reverse)

CCND1
5’-AAGCTCAAGTGGAACCT-3’ (forward)
5’-AGGAAGTTGTTGGGGC-3’ (reverse)

CXCL12
5’-TCAGCCTGAGCTACAGATGC-3’ (forward)
5’-CTTTAGCTTCGGGTCAATGC-3’ (reverse)

TFF1 pre-mRNA
5’-ACCATGGAGAACAAGGTGATC-3’ (forward)
5’-TAAAACAGTGGCTCCTGGCG-3’ (reverse)

GREB1 pre-mRNA
5’-GATAAAAGCAACGTGCGTCTC-3’ (forward)
5’-TCTTGCACAATTCCATCGAG-3’ (reverse)

CCND1 pre-mRNA
5’-CCTACTTCAAATGTGTGCAGAAG-3’ (forward)
5’-CAACAAGTTGCAGGGAAGTC-3’ (reverse)

PgR pre-mRNA
5’-CAGGTCTACCCGCCCTATCT-3’ (forward)
5’-TATATTCTGCGCCCACCTTC-3’ (reverse)

GAPDH
5’-TCTGGTAAAGTGGATATTGTTG-3’ (forward)
5’-GATGGTGATGGGATTTCC-3’ (reverse)

Sequences of siRNAs

siNS
5’-UUCUCCGAACGUGUCACGUdTdT-3’ (sense)
5’-ACGUGACACGUUCGGAGAAdTdT-3’ (anti-sense)

siMLL1
5’-GAUUCGAACACCCAGUUAUdTdT-3’ (sense)
5’-AUAACUGGGUGUUCGAAUCdTdT-3’ (anti-sense)

siMLL1(#2)
5’-GCACUGUUAAACAUUCCACdTdT-3’ (sense)
5’-GUGGAAUGUUUAACAGUGCdTdT-3’ (anti-sense)

siFOXA1
5’-GAGAGAAAAAAUCAACAGCdTdT-3’ (sense)
5’-GCUGUUGAUUUUUUCUCUCdTdT-3’ (anti-sense)

siFOXA1(#2)
5’-GCGAAGUUUAAUGAUCCACdTdT-3’ (sense)
5’-GUGGAUCAUUAAACUUCGCdTdT-3’ (anti-sense)
